# Modelling of the SDF-1/CXCR4 regulated *in vivo* homing of therapeutic mesenchymal stem/stromal cells in mice

**DOI:** 10.7717/peerj.6072

**Published:** 2018-12-06

**Authors:** Wang Jin, Xiaowen Liang, Anastasia Brooks, Kathryn Futrega, Xin Liu, Michael R. Doran, Matthew J. Simpson, Michael S. Roberts, Haolu Wang

**Affiliations:** 1 School of Mathematical Sciences, Queensland University of Technology, Brisbane, Australia; 2 Therapeutics Research Centre, The University of Queensland Diamantina Institute, University of Queensland, Translational Research Institute, Brisbane, Australia; 3 Institute of Health and Biomedical Innovation, Queensland University of Technology, Translational Research Institute, Brisbane, Australia; 4 Mater Research Institute, University of Queensland, Translational Research Institute, Brisbane, Australia; 5 Australian National Centre for the Public Awareness of Science, Australian National University, Canberra, Australia; 6 School of Pharmacy and Medical Science, University of South Australia, Adelaide, Australia

**Keywords:** Mesenchymal stem cells, Stem cell transplantation, Chemotaxis, Mathematical modelling, *In vivo* homing

## Abstract

**Background:**

Mesenchymal stem/stromal cells (MSCs) are a promising tool for cell-based therapies in the treatment of tissue injury. The stromal cell-derived factor-1 (SDF-1)/CXC chemokine receptor 4 (CXCR4) axis plays a significant role in directing MSC homing to sites of injury. However *in vivo* MSC distribution following intravenous transplantation remains poorly understood, potentially hampering the precise prediction and evaluation of therapeutic efficacy.

**Methods:**

A murine model of partial ischemia/reperfusion (I/R) is used to induce liver injury, increase the hepatic levels of SDF-1, and study *in vivo* MSC distribution. Hypoxia-preconditioning increases the expression of CXCR4 in human bone marrow-derived MSCs. Quantitative assays for human DNA using droplet digital PCR (ddPCR) allow us to examine the *in vivo* kinetics of intravenously infused human MSCs in mouse blood and liver. A mathematical model-based system is developed to characterize *in vivo* homing of human MSCs in mouse models with SDF-1 levels in liver and CXCR4 expression on the transfused MSCs. The model is calibrated to experimental data to provide novel estimates of relevant parameter values.

**Results:**

Images of immunohistochemistry for SDF-1 in the mouse liver with I/R injury show a significantly higher SDF-1 level in the I/R injured liver than that in the control. Correspondingly, the ddPCR results illustrate a higher MSC concentration in the I/R injured liver than the normal liver. CXCR4 is overexpressed in hypoxia-preconditioned MSCs. An increased number of hypoxia-preconditioned MSCs in the I/R injured liver is observed from the ddPCR results. The model simulations align with the experimental data of control and hypoxia-preconditioned human MSC distribution in normal and injured mouse livers, and accurately predict the experimental outcomes with different MSC doses.

**Discussion:**

The modelling results suggest that SDF-1 in organs is an effective *in vivo* attractant for MSCs through the SDF-1/CXCR4 axis and reveal the significance of the SDF-1/CXCR4 chemotaxis on *in vivo* homing of MSCs. This *in vivo* modelling approach allows qualitative characterization and prediction of the MSC homing to normal and injured organs on the basis of clinically accessible variables, such as the MSC dose and SDF-1 concentration in blood. This model could also be adapted to abnormal conditions and/or other types of circulating cells to predict *in vivo* homing patterns.

## Introduction

Mesenchymal stem/stromal cells (MSCs) are excellent candidates for use in tissue repair and regeneration (Fu et al., 2018; Niclis et al., 2017; Squillaro, Peluso & Galderisi, 2016; Zhang et al., 2017). Human MSCs can be harvested from a range of tissues (bone marrow and adipose are common sources) with few ethical issues; and these cells can be expanded in number for use on clinical scales within a short time period (Parekkadan & Milwid, 2010; Rohart et al., 2016; Zhang et al., 2017). Similar to the use of pharmacokinetics for drug development, the aim of elucidating *in vivo* kinetics of MSCs is to predict and enhance their therapeutic potential, as well as to minimize adverse effects. For example, MSC overdose and non-specific targeting can result in vascular obstruction and organ entrapment, which leads to various adverse events such as leg pain, dyspnea or even maldifferentiation in the long term (Boltze et al., 2015; Karussis et al., 2010). Hence understanding *in vivo* kinetics of MSCs becomes a critical step in the development of any new therapeutic agent to establish the optimal dosing regimens and targeting strategies (Jin et al., 2016; Zhao et al., 2011).

**Figure 1:**
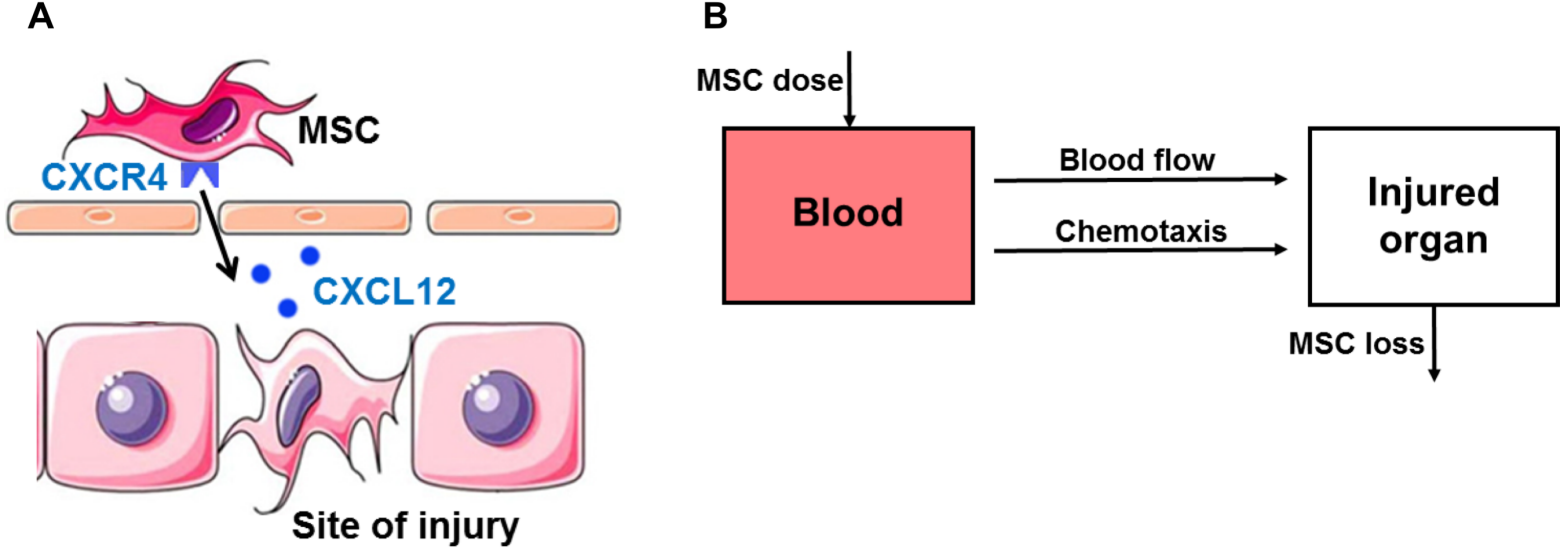
Hypothesis and schematic diagram of modeling *in vivo* homing of therapeutic MSCs. (A) Schematic diagram of the stromal cell-derived factor-1 (SDF-1)/CXC chemokine receptor 4 (CXCR4) axis in *in vivo* homing of MSCs to the sites of hepatic ischemia/reperfusion (I/R) injury. SDF-1 is upregulated at the sites of injury and serves as a potent chemoattractant to recruit circulating or residing MSCs expressing its cognate receptor CXCR4 on the surface. (B) Schematic of compartment model for *in vivo* homing of therapeutic MSCs.

One important mechanism that is often overlooked, but essential for MSC therapy is the homing of MSCs. There are several mediators and receptors involved in the homing of MSCs to sites of injury. A number of studies indicate that the stromal cell-derived factor-1 (SDF-1, also known as CXCL12) is upregulated at sites of injury and serves as a potent chemoattractant to recruit circulating or residing MSCs expressing its cognate receptor CXC chemokine receptor 4 (CXCR4) (Fig. 1A) (Dar et al., 2005; Ji et al., 2004). Although recently CXCL14 and extracellular ubiquitin are found as ligands for CXCR4, SDF-1 is still considered as the most important ligand (Kufareva et al., 2014). The SDF-1/CXCR4 axis promotes stem cell mobilization to injured organs such as brain (Ji et al., 2004), bone (Kitaori et al., 2009), skin (Hu et al., 2013), kidneys (Liu et al., 2012), heart (Abbott et al., 2004) and liver tissues (Kucia et al., 2004). Treating MSCs with hypoxia-preconditioning in culture induces high surface expression of CXCR4 that enhances homing ability (Ji et al., 2004).

In addition to experimental studies, cell kinetics have also been widely studied using various mathematical modelling frameworks to help understand both *in vitro* and *in vivo* mechanisms (Chung et al., 2008; Jin et al., 2016; Werner et al., 2015), and design clinical treatment protocols (Enderling et al., 2009; Werner et al., 2016; Wodarz et al., 2014). In general, there are two types of mathematical models used to study such biological systems: (i) Continuum models that measure population-level properties, such as the concentration or density of populations of cells, without dealing specifically with individual-level properties (Enderling et al., 2009; Jin et al., 2016; Werner et al., 2015; Werner et al., 2016; Wodarz et al., 2014); and (ii) Discrete models that directly simulate individual cells (Holzhütter et al., 2012; Jin et al., 2017). Sometimes, a multi-scale model can be established that predicts both individual- and population-level properties, and this is achieved by taking the continuum limit description of some particular discrete, individual-based model (Jin et al., 2016; Jin, McCue & Simpson, 2018). The first model for the *in vivo* kinetics of MSCs is a population-level model, published in 2016 (Wang et al., 2016). This physiologically-based pharmacokinetic model can characterize and predict the organ distribution of administered MSCs. However, the model neglects effects of tissue injury on MSC distribution, especially the details of chemoattractant cell-adhesion and transmigration mechanisms (Wang et al., 2016; Zhu et al., 1996). As a result, the model underestimates the MSC doses in injured organs.

In this work we develop a mathematical model-based system to characterize the *in vivo* homing of administered human bone marrow-derived MSCs with SDF-1 levels in liver and CXCR4 expression on the transfused MSCs. This continuum model presented here is novel since it includes both passive and active homing mechanisms. We refer to the entrapment of MSCs in small-diameter blood vessels as passive homing, and define active homing as MSCs actively moving to tissues using chemoattractant cell-adhesion and transmigration mechanisms (Karp & Teo, 2009). The model shows good agreement with experimental data, and provides insights into passive and active homing mechanisms. The calibrated model also accurately predicts outcomes with different MSC doses. This *in vivo* modelling approach enables qualitative characterization and prediction of the MSC homing to normal and injured organs.

## Materials and Methods

### Hepatic ischemia-reperfusion (I/R) injury model

All animal procedures are approved by the Animal Ethics Committee of the University of Queensland (MED/493/15/NHMRC) and are carried out in accordance with Australian Code for the Care and Use of Animals for Scientific Purposes 8th edition. Healthy mice (Male 20-week-old BALB/c nude) are anaesthetized initially by an intraperitoneally injection of ketamine hydrochloride (80 mg/kg) and xylazine (10 mg/kg). Body temperature is controlled by placing mice on a heating pad set to 37 °C. Hepatic I/R injury is induced by clamping the portal vein and hepatic artery supplying the median and left lobes using a microvascular clamp. After 45 min of partial ischemia, the clamp is removed to allow reperfusion in the liver.

### Hypoxia-precondition of human MSCs

Bone marrow aspirates are collected from fully informed healthy human volunteer donors who provided written consent. The healthy volunteer donors are recruited from Mater Private Hospital, Brisbane, Australia. Ethical approval is granted through the Mater Health Services Human Research Ethics Committee and ratified by the Queensland University of Technology Human Ethics Committee (number: 1000000938). Human MSCs are isolated from bone marrow aspirates, cultured and characterized as we previously described (Parekkadan & Milwid, 2010; Squillaro, Peluso & Galderisi, 2016). All cells are cultured in monolayer using expansion media formulated from low glucose DMEM (ThermoFischer) supplemented with 10% fetal bovine serum (FBS; Thermo Fisher, Waltham, MA, USA) and 10 ng/mL FGF-1 (Peprotech). All experiments involving MSCs are performed at passage 4-8, tested negative for mycoplasma contamination, and <80% confluence. MSCs are cultured in a hypoxia chamber incubator (catalog No. 27310; StemCell Technologies, Vancouver, BC, Canada) at 37 °C in 3% O_2_, 5% CO_2_ and 92% N_2_ for 24 h, and these MSCs are named as hypoxia-preconditioned MSCs. MSCs cultured for 24 h in 95% air and 5% CO_2_ are used as a control.

### *In vivo* transplantation of MSCs

Male 20-week-old BALB/c nude mice are purchased from the Animal Resource Centre (Perth, Western Australia). 150 µl of a suspension of 5 × 10^5^ or 1. 5 × 10^6^ MSCs is injected with a 27-gauge needle through the tail vein of the control mice or mice with hepatic I/R injury at the time of reperfusion. Prior to injection, the MSCs are maintained at 4 °C, and the cells are gently resuspended with a pipette to ensure no aggregation before injection. Animals (*n* = 3) are sacrificed at designated times (30 min, 4, 15, 24, and 48 h post-injection). Here *n* indicates the number of mice used, following the guidelines for the welfare and use of animals in pharmacokinetic studies (Workman et al., 2010). Blood is obtained by cardiac puncture. The normal liver and the liver with I/R injury are removed for analysis.

### Droplet digital PCR assays for Alu sequences

Genomic DNA (gDNA) of the blood and liver are isolated using DNA Mini Kit (Qiagen, Valencia, CA, USA). Droplet digital PCR (ddPCR) is performed in reaction consisting of gDNA, primer sets (Alu forward: GCCTGTAATCCCAGCACTTT; Alu reverse: CACTACGCCCGGCTAATTT) (Zhao et al., 2011), H_2_O and ddPCR EvaGreen Supermix (BioRad, USA). ddPCR is performed according to manufacturer’s manual. Briefly, 20 µL of ddPCR reaction mix is separated into droplets with QX200 Droplet Generator (BioRad, USA). The droplets are transferred into a 96-well PCR plate, sealed and incubated at following cycling conditions: one cycle of 95 °C for 5 min, 45 cycles of 95 °C for 30 s, 55 °C for 1 min and one cycle of 4 °C for 5 min, 90 °C for 5 min and an infinite hold of 12 °C. After thermal cycling, the PCR plate is transferred in QX200 Droplet Reader (read) and read in FAM channel using QuantaSoft version 1.7.

### Quantitative ELISA analysis

Liver samples are weighed and immediately placed in 10 volumes (wt/vol) of a protease inhibitor cocktail containing 10 nmol/l EDTA, 2 mmol/l PMSF, 0.1 mg/ml soybean trypsin inhibitor, 1.0 mg/ml bovine serum albumin, and 0.002% sodium azide in isotonic PBS, pH 7.0. Tissues are disrupted with a tissue homogenizer, and lysates are incubated at 4 °C for 2 h. Samples are clarified by two rounds of centrifugation at 12,500 g for 10 min at 4 °C. SDF-1 concentrations in blood and liver are assessed by enzyme-linked immunosorbent assay (CUSABIO, TX, USA). CXCR4 concentration in human MSCs are assessed by ELISA (CUSABIO, TX, USA).

### Immunohistochemistry for SDF-1 and CXCR4 expression

Liver tissues and human MSCs are fixed in 10% neutral-buffered formalin, processed, and then embedded in paraffin for light microscopy. Immunohistochemistry is performed following the standard avidin/streptavidin-biotin peroxidase methods. All slides (4 µM) are deparaffinized, rehydrated and boiled for antigen retrieval (30 m at 98 °C in citrate buffer pH 6.0). Primary antibodies against SDF-1 (1:200) and CXCR4 (1:400) proteins (Abcam, USA) are used on the sections of the tumor tissue. After being incubated overnight at 4 °C, the slides are incubated with biotinylated anti-rabbit immunoglobulin for 30 min and then with horseradish peroxidase-jugated streptavidin for 30 min. Negative control experiments include omission of either the primary or secondary antibody with 1% BSA-PBS. Jurkat cells are used as a positive control of SDF-1 expression, and HeLa cells are used as a positive control of CXCR4 expression according to the manufacturer’s instructions. Each step is followed by a washing with PBS. Staining is revealed by 3,3′-diaminobenzidine and counterstained with hematoxylin.

### Model formulation

The population-level mathematical model includes the descriptions of MSC and SDF-1 kinetics in the blood and liver. After intravenous injection, MSCs are arrested in the liver from blood by both passive homing (via blood flow) and active homing (via the liver SDF-1 attracting CXCR4 in MSCs) (Karp & Teo, 2009). The number of MSCs in the liver can decrease due to a series of mechanisms including release back to the blood circulation and depletion. Here we refer to depletion as the loss of cell functionality and viability caused by various mechanisms (Oh, Lee & Wagers, 2014; Wang et al., 2016). Therefore, the governing differential equation describing MSCs in the liver is as follows:

(1) \begin{eqnarray*} \frac{\mathrm{d}{M}_{\mathrm{L}} \left( t \right) }{\mathrm{d}t} & =& \overbrace{\alpha {M}_{\mathrm{B}} \left( t \right) {}}^{\text{Passive homing} \left( \text{via blood flow} \right) }+\overbrace{\beta {S}_{\mathrm{L}} \left( t \right) \left( 1- \frac{{M}_{\mathrm{L}} \left( t \right) }{K} \right) {}}^{\text{Active homing} \left( \text{via SDF}-1/\text{CXCR}4 \right) }\nonumber\\\displaystyle & -& \overbrace{\gamma {M}_{\mathrm{L}} \left( t \right) {}}^{\text{Loss due to release and depletion}}\end{eqnarray*}

where *M*_L_(*t*) (cell/kg) is the dose of MSCs in the liver, *M*_B_(*t*) (cell/kg) is the dose of MSCs in the blood, *S*_L_(*t*) (pg/mL) is the concentration of SDF-1 in the liver, *t* (h) is time, *α* (h^−1^) is the MSC arrest rate associated with blood flow, *β* (cell mL/(kg h pg)) is the MSC arrest rate associated with SDF-1/CXCR4 attraction, *K* (cell/kg) is the attraction capacity of MSCs expressing CXCR4 attracted by SDF-1 in the liver, and *γ* (h^−1^) is the MSC loss rate in the liver including release and depletion.

For MSCs in the blood, a relatively fast dose-decrease at early time, known as the distribution phase, is followed by a slower decrease at later time, known as the elimination phase. These processes can be modelled using a biexponential decay model (Armitage, Dick & Bourne, 2003):

(2) \begin{eqnarray*}{M}_{\mathrm{B}} \left( t \right) =\overbrace{{C}_{1}{\mathrm{e}}^{-{\lambda }_{1}t}{}}^{\text{Distribution phase}}+\overbrace{{C}_{2}{\mathrm{e}}^{-{\lambda }_{2}t}{}}^{\text{Elimination phase}}\end{eqnarray*}

where *C*_1_ (cell/kg) and *C*_2_ (cell/kg) are the intercepts for the distribution and elimination phases of MSCs, and *λ*_1_ (h^−1^) and *λ*_2_ (h^−1^) are the decay rates for the distribution and elimination phases of MSCs, respectively.

In normal mice, the SDF-1 concentration in the blood remains approximately constant. For SDF-1 in the blood with an injured liver, the initial concentration is the same as that of normal uninjured mice and increases at early reperfusion followed by a relatively slower decrease at later time. Therefore, the SDF-1 in the blood with an injured organ is modelled using the function form associated with modified-biexponential decay (Wilson et al., 2015):

(3) \begin{eqnarray*}{S}_{\mathrm{B}}(t)=\overbrace{{S}_{\mathrm{B}} \left( 0 \right) {}}^{\text{Initial SDF}-1 \text{concentration}}+\overbrace{{a}_{\mathrm{B}}{\mathrm{e}}^{-{b}_{\mathrm{B}}t} \left( 1-{\mathrm{e}}^{-{c}_{\mathrm{B}}t} \right) {}}^{\text{Kinetics of SDF}-1}\end{eqnarray*}

where *S*_B_(0) (pg/mL) is the initial concentration of SDF-1 in the blood of mice with injured liver, *a*_B_ (pg/mL) is the amplitude of SDF-1 concentration change, *b*_B_ (h^−1^) is the SDF-1 decay rate, and *c*_B_ (h^−1^) is the control factor of SDF-1 kinetics.

In normal mice, the SDF-1 concentration in the liver remains approximately constant. SDF-1 concentration in the injured liver has the same function form as in the blood:

(4) \begin{eqnarray*}{S}_{\mathrm{L}}(t)={S}_{\mathrm{L}}(0)+{a}_{\mathrm{L}}{\mathrm{e}}^{-{b}_{\mathrm{L}}t} \left( 1-{\mathrm{e}}^{-{c}_{\mathrm{L}}t} \right) \end{eqnarray*}

where *S*_L_ (0), *a*_L_= *a*_B_/*η*_1_, *b*_L_= *b*_B_/*η*_2_, and *c*_L_ = *c*_B_/*η*_3_ are the corresponding parameters in the liver that have the same physiological meanings as described in the model for SDF-1 kinetics in the blood. To develop the model on the basis of clinically accessible variables, the parameters for the SDF-1 in the liver are presented in terms of their relations with the corresponding parameters in the blood by association coefficients *η*_1_, *η*_2_, and *η*_3_.

### Model calibration

The calibration of the model is performed using MATLAB’s nonlinear curve-fitting function, *lsqcurvefit* (MathWorks, 2018). Both models for SDF-1 and MSCs in the blood are calibrated with experimental data. The association coefficients *η*_1_, *η*_2_, and *η*_3_ are then determined by comparing the calibrated models for SDF-1 in the blood and liver, and are validated by predicting the SDF-1 concentration in the liver based on the calibrated model for the SDF-1 concentration in the blood using published independent external data (Wilson et al., 2015). Details of the validation of the association coefficients are shown in the Supplemental Information. The calibrated models for SDF-1 in the liver and MSCs in the blood are then inputted into the model for MSCs in the liver, to estimate the parameters in normal and injured livers, respectively.

**Figure 2:**
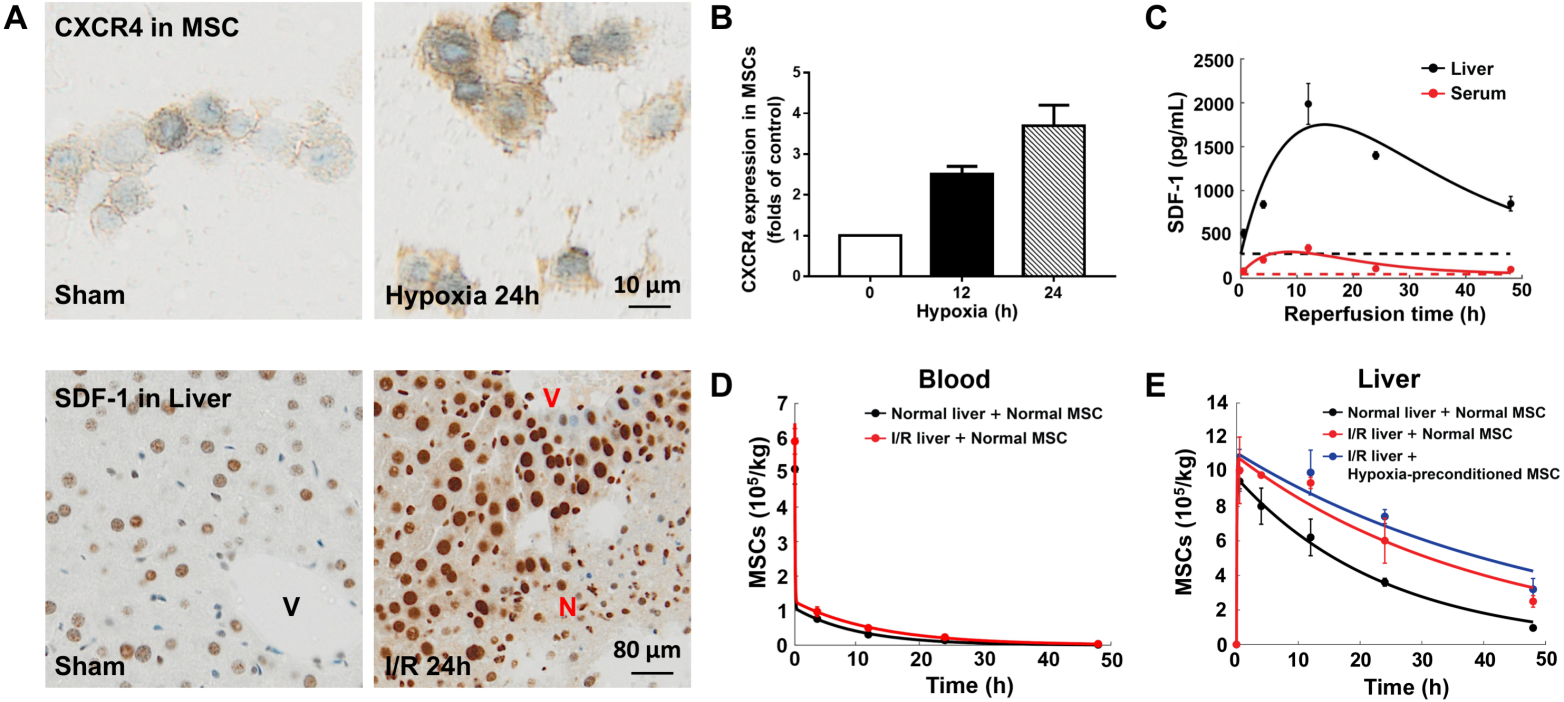
Model calibration results with experimental data. (A) Representative micrographs of immunohistochemistry for CXCR4 in hypoxia-preconditioned MSCs (3% O_2_) and SDF-1 in mouse liver with ischemia/reperfusion (I/R) injury. (B) CXCR4 levels in control MSCs and hypoxia-preconditioned MSCs (3% O_2_). Quantitative ELISA was used for the analysis of CXCR4 levels in MSCs. (C) Model calibration with the SDF-1 concentrations in the blood and liver of mice with hepatic ischemia/reperfusion (I/R) injury. (D) Model calibration with the MSC concentrations in the blood of normal mice and mice with hepatic I/R injury at dose of 5 × 10^5^ cells/animal. (E) Model calibration with the normal and hypoxia-preconditioned MSC concentrations in the liver of normal mice and mice with hepatic I/R injury at dose of 5 × 10^5^ cells/animal. The solid line in each panel represents the concentration-time profile of the SDF-1 and MSCs simulated by the model while the circles represent measured data. Concentrations of the SDF-1 and MSCs are expressed as SDF-1 amount and number of cells per kilogram of tissue. The data are expressed as the sample mean ± one sample standard deviation.

## Results

### Experimental results

Previous studies show that SDF-1 expression increases in the liver with ischemia/reperfusion (I/R) injury (Lentsch et al., 1999; Wilson et al., 2015). Our immunohistochemistry for SDF-1 in the mouse liver with ischemia/reperfusion (I/R) injury also reveals a significantly higher SDF-1 level in the I/R injured liver than the control (Figs. 2A and 2C). It has been reported that SDF-1 can activate two chemokine receptors, CXCR4 and CXCR7, with different downstream signaling pathways during liver injury (Lentsch et al., 1999; Wilson et al., 2015). To elucidate the SDF-1/CXCR4 regulated *in vivo* homing of human MSCs, we use droplet digital PCR assays for human-specific Alu sequences to quantify the numbers MSCs in the blood and liver of normal and hepatic I/R injured mice (Figs. 2D and 2E). Indeed, a higher MSC concentration is found in the I/R injured liver (Fig. 2E).

In conjunction with Figs. 2A, 2B shows the overexpression of CXCR4 in hypoxia-preconditioned MSCs, which is consistent with previous experimental observations (Cencioni, Capogrossi & Napolitano, 2012; Liu et al., 2012). Comparing with the normal MSCs, the CXCR4 expression in 24 h hypoxia-preconditioned MSCs is about 4-fold higher. Previous studies find that the hypoxia preconditioned MSCs also become more active in terms of both cell motility and proliferation (Ali et al., 2016; Beegle et al., 2015; Liu et al., 2012). Aligning with these experimental findings, our ddPCR results show an increased number of hypoxia-preconditioned MSCs in the I/R injured liver comparing to the normal MSCs in the same liver condition. However, at this stage how the hypoxia-preconditioning facilitates *in vivo* homing of MSCs remains unclear. For example, it is unclear whether the hypoxic preconditioning enhances MSC homing through: (i) the SDF-1/CXCR4 chemotaxis (active homing); or (ii) the transportation via blood flow (passive homing); or (iii) a combination of effects from (i) and (ii). We now attempt to distinguish between these two possibilities by calibrating our mathematical model to the experimental data.

### Modelling results

The mathematical model for *in vivo* human bone marrow-derived MSC homing is developed based on the published intravital imaging details of administered MSCs (Toma et al., 2009; Wang et al., 2016) and SDF-1/CXCR4 chemotaxis of MSCs (Fig. 1A) (Dar et al., 2005; Ji et al., 2004). Following intravenous injection, MSCs are arrested in organs by both passive homing (via blood flow) and active homing (via the organ SDF-1 attracting CXCR4 expressing MSCs) (Karp & Teo, 2009). MSC release and depletion in organs are described by a single loss term in our model. Differentiation is not included in the model as differentiation is slow relative to the time scale of the experiment and hence would have a small impact on the MSC distribution at the organ level over the observation period (Hass et al., 2011; Schmidt et al., 2006). As shown in Fig. 1B, this model has two compartments: blood and injured organ (liver). All MSCs are assumed to act independently with no obligatory connections or intercellular feedback loops. In summary, we assume the *in vivo* kinetics of MSCs are governed by two processes: (i) transport to the organ (liver) via blood flow and the SDF-1/CXCR4 chemotaxis; and (ii) loss in the organ by release and depletion. Variables included in the model are time *t* (h), SDF-1 concentration in blood *S*_B_(*t*) (pg/mL) and liver *S*_L_(*t*) (pg/mL), and MSC dose in blood *M*_B_(*t*) (cell/kg) and liver *M*_L_(*t*) (cell/kg).

Previous modelling of the *in vivo* homing of MSCs in organs neglects SDF-1/CXCR4 chemotaxis (Wang et al., 2016), while the biological evidence suggests that this mechanism plays an important role in the MSC homing (Abbott et al., 2004; Hu et al., 2013; Lentsch et al., 1999; Wilson et al., 2015). With the SDF-1/CXCR4 chemotaxis incorporated in our model, we calibrate the model system (Eqs. 1–4) to the experimental data, as shown in Figs. 2C–2E. The model captures key features of the observed time evolution of MSC dose in the mouse liver with a high goodness-of-fit, with *R*^2^=0.987 (Fig. S1). The SDF-1 profiles in Fig. 2C show that the SDF-1levels in the liver and blood are maximally increased after approximately 12 h of reperfusion, which correlates with maximal liver injury after hepatic I/R injury reported in previous studies (Lentsch et al., 1999; Wilson et al., 2015). Following intravenous injection, the MSC dose in the liver increases until 4 h after injection, and then slowly declined. The area under the curve (AUC_0−48hr_) of MSCs in the liver indicates that organ loading of MSCs (Fig. 2E) increases by 1.52 times following hepatic I/R injury (from 2.00 × 10^9^cells h/kg to 3.05 × 10^9^ cells h/kg), and the organ loading of hypoxia-preconditioned MSCs (Fig. 2E) increases by 1.71 times (3.43 × 10^8^ cells h/kg). The increased organ loading suggests that the injured liver is an effective attractant for both normal and hypoxia-preconditioned MSCs.

**Table 1:** Values and reference of parameters in the model.

Parameter (unit)	Description	Dimensional			Reference
		Normal	I/R	I/R with hypoxia- preconditioning	
*S*_B_ (0) (pg/mL)^a^	Initial SDF-1 in blood	48	–	–	Karp & Teo (2009) and measured
*a*_B_ (pg/mL)^b^	Amplitude of SDF-1 concentration change	N/A	7. 94 × 10^4^	–	Estimated
*b*_B_ (h^−1^)	SDF-1 decay rate	N/A	0.11	–	Estimated
*c*_B_ (h^−1^)	Control factor of SDF-1 kinetics	N/A	0.001	–	Estimated
*η*_1_	Association coefficient	N/A	0.30	–	Estimated
*η*_2_	Association coefficient	N/A	1.73	–	Estimated
*η*_3_	Association coefficient	N/A	1.00	–	Estimated
*S*_L_ (0) (pg/mL)^a^	Initial SDF-1 in liver	278	–	–	Karp & Teo (2009) and measured
*C*_1_(cell/kg)^a^	Intercept for the distribution phase of MSCs	2.94 × 10^9^ (5 × 10^5^ dose)	–	–	Estimated
		8.82 × 10^9^ (1.5 × 10^6^ dose)	–	N/A	
*C*_2_ (cell/kg)^b^	Intercept for the elimination phase of MSCs	1.12 × 10^5^ (5 × 10^5^ dose)	1. 31 × 10^5^ (5 × 10^5^ dose)	–	Estimated
		3.59 × 10^5^ (1.5 × 10^6^ dose)	3.38 × 10^5^ (1.5 × 10^6^ dose)	N/A	
*λ*_1_ (h^−1^)^a^	Slope of the distribution phase of MSCs	17.52	–	–	Estimated
*λ*_2_ (h^−1^)^b^	Slope for the elimination phase of MSCs	0.10 (5 × 10^5^ dose)	0.08 (5 × 10^5^ dose)	–	Estimated
		0.07 (1.5 × 10^6^ dose)	0.04 (1.5 × 10^6^ dose)	N/A	
*α* (h^−1^)^a^	MSC arrest rate associated with blood flow	0.64	0.71	0.72	Wang et al. (2016) and estimated
*β* (pg^a^h)	MSC arrest rate associated with SDF-1/CXCR4 attraction	0.01	0.12	0.19	Estimated
*γ* (h^−1^)	MSC loss rate in organ	0.04	0.03	0.02	Wang et al. (2016) and estimated
*K* (cell/kg)	SDF-1/CXCR4 attraction capacity in organ	4. 63 × 10^6^	5.29 × 10^6^	2. 20 × 10^7^	Estimated

**Notes.**

a Same value for all organ and MSC conditions.

b Same value for all MSC conditions.

The parameter estimates obtained by calibrating the model to match the experimental data are listed in Table 1. These parameter estimates reveal three important features:

1. Estimates of *α*, which represents the MSC arrest rate associated with blood flow, are approximately the same for all liver and MSC conditions. The small change (about 10%) in *α* estimates suggests that neither the liver nor MSC conditions have significant impact on the passive homing,
2. The highest SDF-1/CXCR4 attraction capacity and MSC arrest rate associated with SDF-1/CXCR4 attraction are obtained for the hypoxia-preconditioned MSCs in I/R injured livers. Both the SDF-1/CXCR4 attraction capacity and the corresponding MSC arrest rate significantly increase (over 100%) compared to those obtained for the normal MSCs in normal livers, indicating that SDF-1 in organs is an effective *in vivo* attractant for MSCs expressing CXCR4,
3. The MSC depletion rate is lower for the hypoxia-preconditioned MSCs than for the untreated MSCs, which is consistent with results from previous studies that hypoxic preconditioning enhances the MSC survival *in vivo* (Beegle et al., 2015; Liu et al., 2012). Based on our modelling results, we suggest that the hypoxic preconditioning enhances *in vivo* homing of MSCs though active homing and the survival of MSCs in the organ, whereas its impact on passive homing is small.

**Figure 3:**
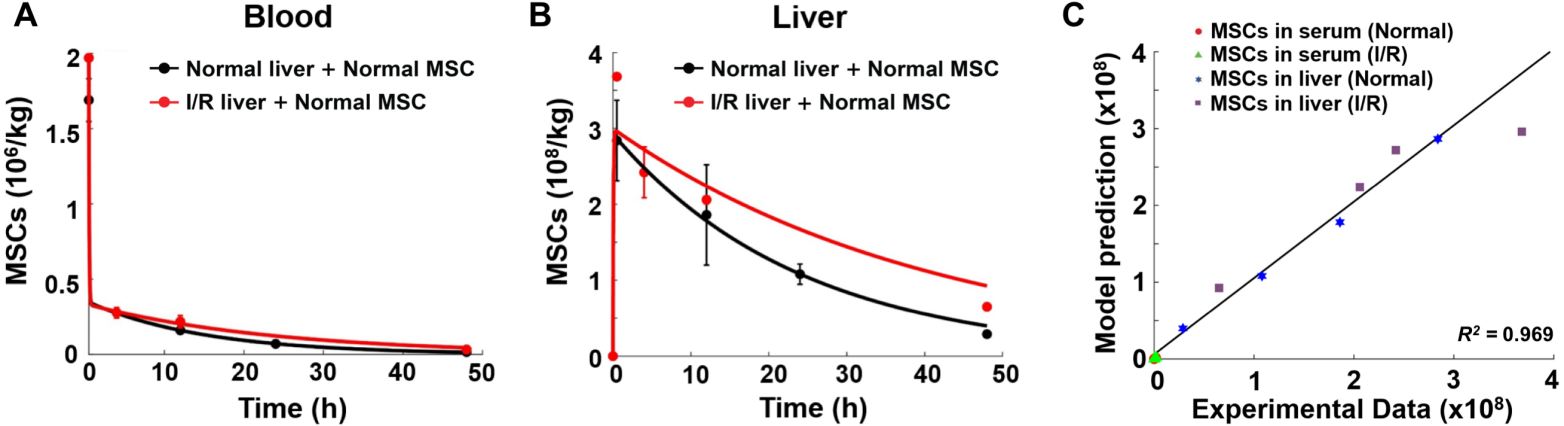
Model validation results with experimental data. (A) Model validation with the MSC concentrations in the blood of normal mice and mice with hepatic I/R injury at a dose of 1.5 × 10^6^ cells/animal. (B) Model validation with the MSC concentrations in the liver of normal mice and mice with hepatic I/R injury at a dose of 1.5 × 10^6^ cells/animal. The solid line in each panel represents the concentration-time profile of the MSCs simulated by the model while the circles represent measured data. Concentration of the MSCs is expressed as the number of cells per kilogram of tissue. The data are expressed as the mean ± one standard deviation. (C) Goodness-of-fit plot of model validation. Model predictions and experimental data were analyzed using linear regression, with *R*^2^ = 0.969 (*n* = 16).

To further validate the model, simulations of SDF-1 levels in mouse blood and livers are compared with published data (Wilson et al., 2015). All parameters are obtained using the same approach described in the Methods section. As shown in Fig. S2, there is a high goodness-of-fit with *R*^2^=0.986, between model predictions and the independent data, indicating that the model is suitable to characterize the *in vivo* kinetics of SDF-1. Our model is then used to predict the *in vivo* homing of the MSCs administered at a different initial dose (1.5 × 10^6^ cells/animal). All parameters in the model are maintained the same as shown in Table 1, and we find that the model adequately predicts the MSC doses in both normal and injured livers, again with a high goodness-of-fit with *R*^2^ close to unity (Fig. 3). The good agreement between the model predictions and experimental data confirms that this model can be readily applied to different MSC dose regimens. There is substantial evidence that administered MSCs would accumulate within sites of disease or injury (Hu et al., 2013; Ji et al., 2004; Kitaori et al., 2009; Liu et al., 2012). However, previously published cytokinetic models often underestimate the therapeutic cell concentration in diseased organs such as the heart with myocardial infarction or fibrotic liver (Wang et al., 2016; Zhu et al., 1996). As our model includes the important SDF-1/CXCR4 axis which regulates the *in vivo* homing of stem cells to sites of injury, it is able to account for the effect of tissue injury on MSC distribution.

## Discussion

There is a growing interest in MSCs in the context of regenerative medicine for treating injured organs (Fu et al., 2018; Squillaro, Peluso & Galderisi, 2016; Zhang et al., 2017). Therefore, understanding the kinetics, including the homing of MSCs, is becoming crucial to improve treatment outcomes. Previous studies find that the SDF-1/CXCR4 axis is important in the homing of MSCs to injured organs, and help mobilization of the MSCs to injured tissues (Hu et al., 2013; Ji et al., 2004; Kitaori et al., 2009; Liu et al., 2012; Wilson et al., 2015). Besides, studies have also reported that MSCs are inherently tumor-homing including lung, brain, breast, colon, and ovarian carcinomas (Reagan & Kaplan, 2011). SDF-1 is one of the identified tumor-derived factors which can motivate MSC tumor migration (Gao et al., 2009; Klopp et al., 2007). The first physiologically-based pharmacokinetic model for the *in vivo* kinetics of MSCs characterizes and predicts the organ distribution of administered MSCs (Wang et al., 2016). However, the model assumes that MSCs arrest into organs mainly due to the blood flow. To the best of our knowledge, there are no mathematical models that capture features of the SDF-1/CXCR4 chemotaxis in injured organs at present. In this study we develop a mathematical model to characterize *in vivo* homing of administered human bone marrow-derived MSCs. The model considers both MSC and SDF-1 kinetics in the blood and organ and assumes that MSCs arrest in organs via both passive homing through blood flow, and active homing through the organ SDF-1 attracting CXCR4 on MSCs.

The previous mathematical model underestimates the MSC doses in the injured livers (Wang et al., 2016), due to its large scale and the neglect of detailed active homing mechanisms. Our calibrated mathematical model captures the key features of the experimental data sets. Comparing the parameter estimates for different cases illustrates that the liver and MSC conditions have small impact on the passive homing mechanism. On the other hand, the hypoxia-preconditioned MSCs result in a higher arrest rate associate with the SDF-1/CXCR4 chemotaxis and a lower loss rate, and therefore lead to a higher MSC dose in the liver. As the hypoxia-preconditioned MSCs are characterized by the overexpressed CXCR4, our modelling results reveal the significance of the SDF-1/CXCR4 axis. The calibrated model also well predicts the MSC dose initiated with a different amount. The model developed in this work is the first one that describes and quantifies *in vivo* homing of MSCs via both passive and active mechanisms. Although there is a lack of similar measured or estimated parameters in the literature to compare with, the model provides insights into the impacts of SDF-1/CXCR4 axis on *in vivo* MSC homing through the comparison of the parameter estimates for different liver and MSC conditions. To further validate the experimental observations, the number of mice used in the *in vivo* experiments could be increased. Since previous studies show that MSCs undergo similar processes arresting into various organs (Abbott et al., 2004; Ji et al., 2004; Kucia et al., 2004; Liu et al., 2012; Squillaro, Peluso & Galderisi, 2016), our model can possibly be generalized to predict the MSC homing, as well as the SDF-1 level in other organs by calibrating the model to other experimental datasets.

In most clinical settings, it is impossible to characterize the number of unlabeled MSCs in organs. Since our model is developed on the basis of clinically accessible variables, such as MSC dose and SDF-1 concentration in blood, it may be further developed to predict the homing of MSCs in human bodies. This model can be more useful for clinical applications because it has a less complicated framework and fewer parameters than the previous ones; and enables a more efficient and rational design of MSC therapies by precise prediction of MSC homing to target organs with injury.

## Conclusion

In summary, through the development of the model that incorporates the critical SDF-1/CXCR4 chemotaxis, we demonstrate that it is possible to predict the *in vivo* distribution of administered MSCs in normal and injured livers using clinically accessible variables. Our study provides proof-of-concept for the novel use of mathematical modelling to study the kinetics of MSCs in normal and injured organs for more efficiently designing stem cell-based therapies.

##  Supplemental Information

10.7717/peerj.6072/supp-1 Supplemental Information 1 Supplemental Information Additional results of model calibration and validation

10.7717/peerj.6072/supp-2 Data S1 Raw data

## Additional Information and Declarations

### Data Availability

The following information was supplied regarding data availability: The raw measurements are available in a Supplemental File. The raw data shows SDF-1 levels, and MSC doses in the blood and liver, with various liver and MSC conditions.
