# Supplementary material for: Modelling of the SDF-1/CXCR4 regulated in vivo homing of therapeutic mesenchymal stem/stromal cells in mice
Source: PeerJ. 2018 Dec 6;6:e6072. doi: 10.7717/peerj.6072 (PMC6286806; doi:10.7717/peerj.6072)
Supplement: Supplemental Information 1 — Additional results of model calibration and validation [file peerj-06-6072-s001.docx]

**Supplemental Information**

**Modelling of the SDF-1/CXCR4 Regulated *In vivo* Homing of Therapeutic Mesenchymal Stem/stromal Cells**

**Wang Jin^a^, Xiaowen Liang^b^, Anastasia Brooks^b^, Kathryn Futrega^c^, Xin Liu^b^, Michael R. Doran^c,d,e^, Matthew J. Simpson^a^, Michael S. Roberts^b,f^, Haolu Wang^b^**

^a^ School of Mathematical Sciences, Queensland University of Technology, Brisbane, Australia

^b^Therapeutics Research Centre, The University of Queensland Diamantina Institute, The University of Queensland, Translational Research Institute, Brisbane, Australia

^c^Institute of Health and Biomedical Innovation, Queensland University of Technology, Translational Research Institute, Brisbane, Australia

^d^Mater Research Institute, The University of Queensland, Translational Research Institute, Brisbane, Australia

^e^Australian National Centre for the Public Awareness of Science, Australian National University, Canberra, Australia

^f^School of Pharmacy and Medical Science, University of South Australia, Adelaide, Australia

Correspondence: Michael S. Roberts, Ph.D., The University of Queensland Diamantina Institute, The University of Queensland, Translational Research Institute, QLD 4103, Australia. Telephone: 61-07-3443-8033; Email: m.roberts@uq.edu.au and Haolu Wang, MBBS., Ph.D., The University of Queensland Diamantina Institute, The University of Queensland, Translational Research Institute, QLD 4103, Australia. Telephone: 61-07-3443-7488; Email: [h.wang21@uq.edu.au](mailto:h.wang21@uq.edu.au)

**Additional results**

Fig. S1 shows the overall goodness-of-fit of the model calibration with an initial MSC does of 5 × 10^5^ cells/animal. Fig. S2 shows the model calibration and validation with the independent external data, as well as the corresponding goodness-of-fit plot. To validate the model for SDF-1 in the liver, we first calibrate Equation (3) in the main manuscript to the published independent external data (Wilson et al., 2016), to estimate parameters *a*_B_, *b*_B_, and *c*_B_. The parameter estimates are then inputted into Equation (4) with the association coefficients $\eta_{1}$, $\eta_{2}$, and $\eta_{3}$ estimated by calibrating both Equations (3) and (4) into the data of SDF-1level presented in the main manuscript, to predict the SDF-1 level in the liver. The predicted SDF-1 level (black solid line in Fig. S2A) is then superimposed onto the independent external data.


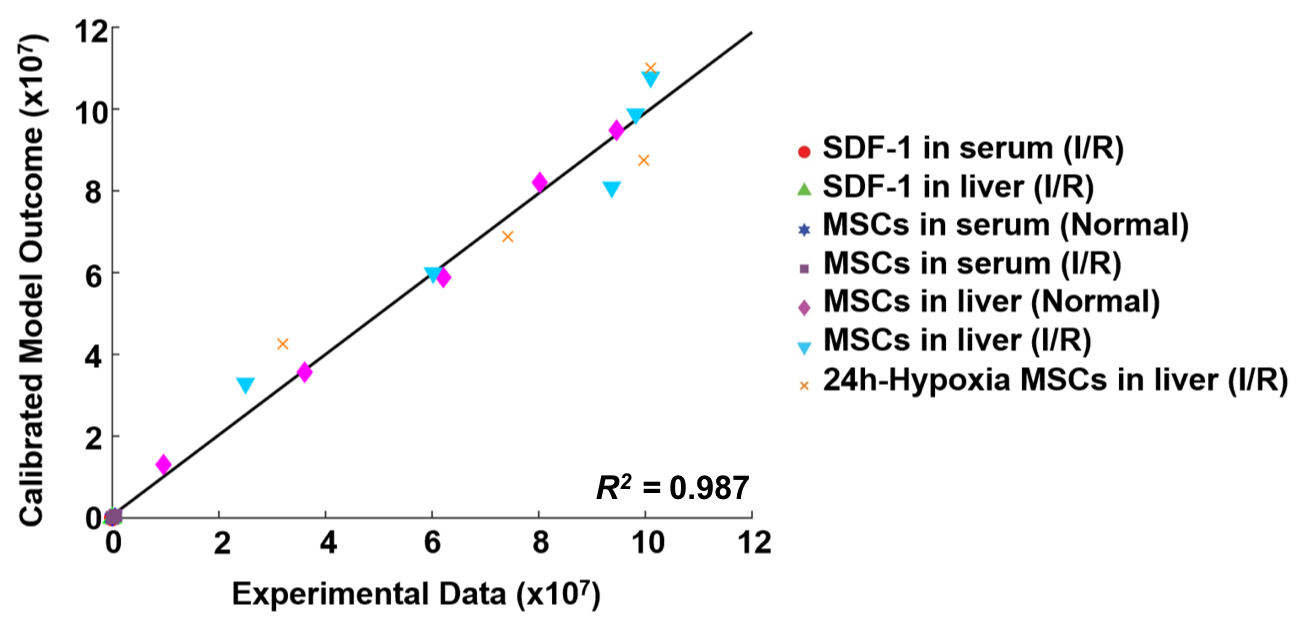


**Figure S1.** Goodness-of-fit plot of model calibration. Model predictions and experimental data are analyzed using linear regression, with *R^2^*=0.987 (n = 34).


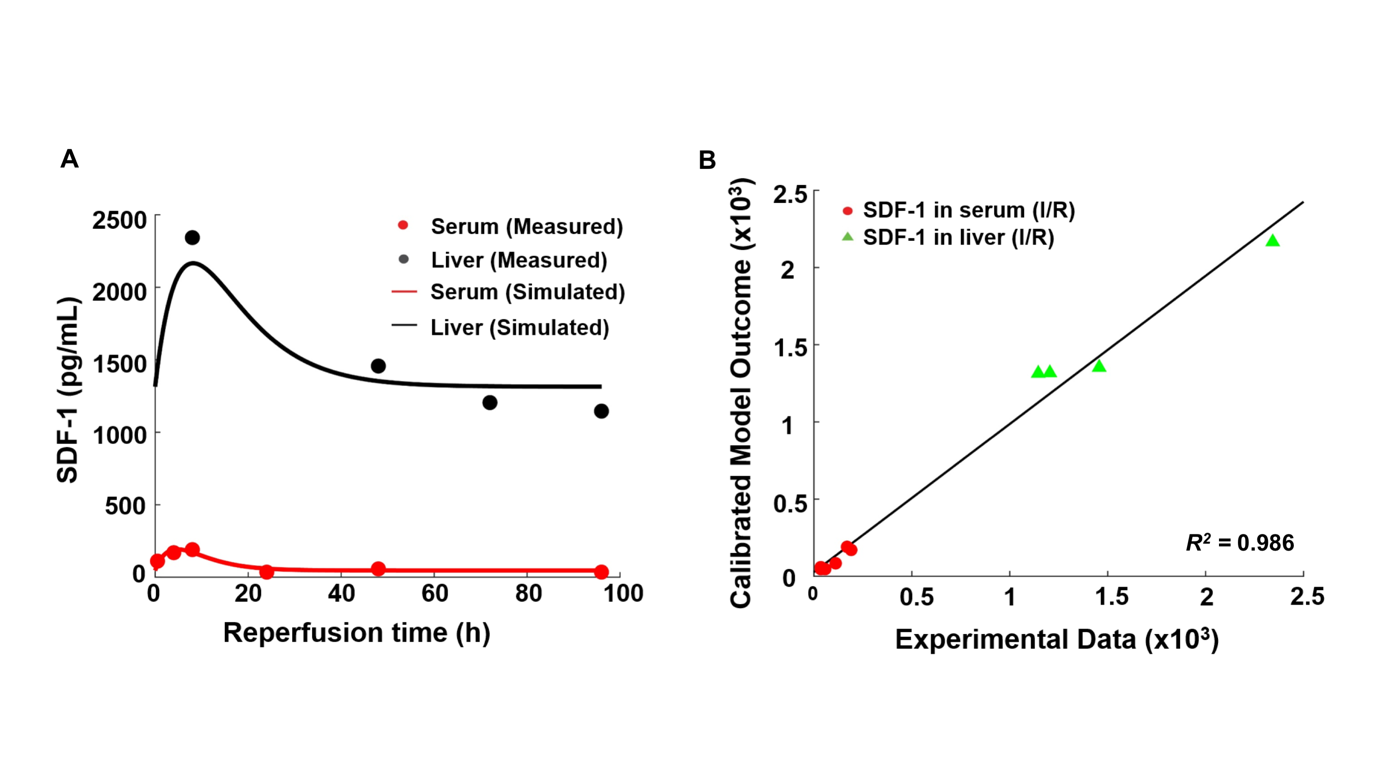


**Figure S2.** Model validation results with independent external data. **(A)** Model validation with the SDF-1 concentrations in the blood and liver of mice with hepatic ischemia/reperfusion (I/R) injury. The solid line in each panel represents the concentration-time profile of the SDF-1 simulated by the model while the circles represent measured data. Concentrations of the SDF-1 is expressed as SDF-1 amount per kilogram of tissue. **(B)** Goodness-of-fit plot of model validation. Model predictions and experimental data are analyzed using linear regression, with *R^2^*=0.986 (n = 10).
